# Spontaneous Coronary Artery Dissection: A Rare Manifestation of Alport Syndrome

**DOI:** 10.1155/2017/1705927

**Published:** 2017-08-14

**Authors:** Amornpol Anuwatworn, Prince Sethi, Kelly Steffen, Orvar Jonsson, Marian Petrasko

**Affiliations:** University of South Dakota Sanford School of Medicine, Sanford USD Medical Center, Sanford Cardiovascular Institute, Sioux Falls, SD, USA

## Abstract

Alport syndrome (AS) is a genetic disorder due to inheritance of genetic mutations which lead to production of abnormal type IV collagen. AS has been associated with renal, auditory, and ocular diseases due to the presence of abnormal alpha chains of type IV collagen in the glomerulus, cochlea, cornea, lens, and retina. The resulting disorder includes hereditary nephritis, corneal opacities, anterior lenticonus, fleck retinopathy, temporal retinal thinning, and sensorineural deafness. Aortic and aortic valve pathologies have been described as extrarenal manifestations of AS in multiple case reports. One case report described intramural hematoma of the coronary artery. We report the first case of true spontaneous coronary artery dissection (SCAD) with an intimal flap as a very rare manifestation of AS. The patient is a 36-year-old female with history of AS with chronic kidney disease, hypertension, and obesity who presented to the emergency room with acute onset of substernal chest pain radiating to her neck and arms. Troponin was elevated, and ECG showed transient 1 mm ST-segment elevation in the inferior leads. Subsequent coronary angiography revealed localized dissection of the left circumflex artery. Percutaneous coronary angioplasty was performed and her symptoms improved. This case illustrates that SCAD may be a manifestation of AS patients with chest pain.

## 1. Introduction

Alport syndrome (AS) is a genetic disorder due to the inheritance of multiple defective genes which lead to production and deposition of abnormal type IV collagen [1]. AS has been associated with renal, auditory, and ocular disease owing to the presence of alpha chains of type IV collagen in the glomerulus, cochlea, cornea, lens, and retina resulting in spectrum of hereditary nephritis [2], corneal opacities, anterior lenticonus, fleck retinopathy, temporal retinal thinning [3], and sensorineural deafness [4]. AS has also been associated with significant aortic disease including dissection and aneurysm [5]. One case of coronary intramural hematoma has been reported [6]. We report the first case of true spontaneous coronary artery dissection (SCAD) with an intimal flap as a very rare manifestation of AS.

## 2. Case Presentation

A 36-year-old woman with X linked AS (*COL4A5*, very rare heterozygous nonsense mutation-c.1117C>T, p.Arg373stop in exon 19), chronic kidney disease, hypertension, and obesity presented to the emergency room with the acute onset of substernal chest and neck pain. Chest pain was associated with exertion. Her pain radiated to both arms and was associated with numbness. She did not have a past history of oral contraceptive pill use and was not pregnant at this time. She was previously prescribed lisinopril for hypertension; however her blood pressure was poorly controlled due to noncompliance in taking her medication over the past month. Her blood pressure was 150/100 mmHg six weeks prior to the admission.

The patient was diagnosed with AS at 23 years of age when she was being considered as a kidney donor for her brother. The diagnosis of AS was confirmed by genetic test and renal biopsy. She was asymptomatic at that time and had no renal, ocular, or auditory manifestations of AS. Later, she developed chronic kidney disease and hypertension. She denied a history of tobacco use/abuse. There was a family history of AS in her brother with AS who underwent two renal transplantations.

On physical exam she had a temperature of 36.3°C, a blood pressure of 152/100 mmHg, a heart rate of 60 beats per minute, and a respiratory rate of 10 breaths per minute. Repeat blood pressure in the ER was 138/88 mmHg. ECG showed transient 1 mm ST-segment elevation in the inferior leads (II, III, and aVF). The serum troponin I was 28 ng/ml (normal range 0–0.01 ng/ml). Serum creatinine was 2.19 mg/dL.

**Figure 1:**
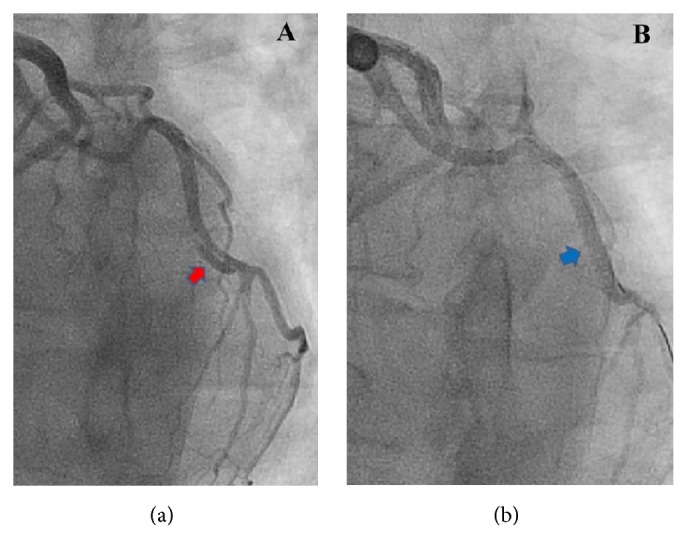
(a) Coronary angiogram shows an intimal flap in the mid left circumflex coronary artery (LCx) confirming coronary artery dissection. (b) Coronary angiogram shows complete revascularization of the LCx after percutaneous coronary intervention. Radiopaque tip of a guide wire was seen in the distal LCx.

Heparin and aspirin were promptly administered. A decision was made to perform emergent coronary angiography, which showed a localized dissection (red arrow in Figure 1(a)) of the mid left circumflex coronary artery. This dissection resulted in a luminal compression of the coronary artery leading to 40% narrowing of its lumen. Percutaneous balloon angioplasty and drug eluting stent placement (blue arrow in Figure 1(b)) were successfully performed. Echocardiography revealed a normal ejection fraction with mild inferolateral wall hypokinesis. She improved clinically with resolution of the chest pain. Following the procedure, she received aspirin, prasugrel, metoprolol tartrate, amlodipine, and atorvastatin. During the hospitalization, her blood pressure was in the range of 118–141/64–89 mmHg.

Six months after procedure she presented to a clinic with a complaint of dyspnea on exertion and atypical chest pain. An exercise stress test with echocardiography did not demonstrate ischemia.

## 3. Discussion

AS is an inherited disorder affecting alpha chains of type IV collagen. There are multiple mutations and modes of inheritance possible including X linked, autosomal recessive, and autosomal dominant [1]. X linked is the most common accounting for approximately 85% of cases with the* COL4A5* mutation being found on the X chromosome [1, 7]. The* COL4A5* gene encodes the *α*5 chain of type IV collagen. These mutations lead to impairment of the production, deposition, and function of collagen type IV alpha chains.

AS has been predominantly associated with renal, auditory, and ocular diseases owing to presence of alpha chains of type IV collagen in the glomerulus, cochlea, cornea, lens, and retina [1]. The *α*5 and *α*6 chains of type IV collagen are also found in the basement membranes surrounding vascular smooth muscle cells in the intima and media of aorta and other arteries in mice model. Seki et al. believed that *α*5 and *α*6 chains of type IV collagen in the basement membranes may have particular function in the arteries which are required to tolerate strong pulse and blood pressure such as the aorta [8]. This may explain the potential mechanism of aortic and coronary complications in AS.

Extrarenal manifestations of AS have been reported with many types of aortic and aortic valve pathologies [5]. AS was associated with aortic abnormalities including aortic dilatation, ruptured ascending aortic aneurysm, aortic dissection, aortic insufficiency, and bicuspid aortic valve in male patients [5, 9]. In a wild-type mouse model, staining for *α*5 chain of type IV collagen was observed in the aorta. On the contrary, no *α*5 chains of type IV collagen in the aortic media were seen on immunostaining in mice with X linked AS due to a nonsense mutation in the* COL4A5* gene. However, there was no aortic pathology seen in these Alport mice. These findings suggested that a lack of *α*5 chain of type IV collagen in the aortic media may be a potential predisposing factor of aortic disease but other contributory variables are needed to produce the clinical manifestation of aortic pathology [5].

Our review of literature has found one case of coronary intramural hematoma in 65-year-old female patient with AS and hypertension who was treated conservatively. We also reviewed another case of giant coronary aneurysms of the left circumflex artery and the right coronary artery in a 50-year-old male with history of AS, hypertension, and smoking presenting with ventricular fibrillation cardiac arrest [10]. In our case, we report the first SCAD with a presence of an intimal flap in the left circumflex artery. Coronary intramural hematoma has been one of the presumed precursors of SCAD [11].

SCAD is typically described as a nontraumatic separation of coronary arterial wall that is not caused by an atherosclerotic process [11, 12]. It has been associated with multiple connective tissue disorders including Marfan syndrome, Ehler-Danlos syndrome, fibromuscular dysplasia, Loeys–Dietz syndromes, and systemic lupus erythematosus. In addition it occurs in multisystem inflammatory or immunologic conditions like rheumatoid arthritis, polyarteritis nodosa, Crohn's disease, sarcoidosis, and polycystic kidney disease [13, 14]. Moreover, SCAD is associated with the pregnant and postpartum states, vigorous physical exercise, blunt chest trauma, drug abuse (like cocaine), and hormonal therapy.

Local stress and shearing may be important contributing factors contributing to the development of SCAD [13]. Vulnerable coronary vessel walls have been proposed as potential mechanism of SCAD. For instance, in pregnancy and postpartum related SCAD, hemodynamic stress due to increase in blood volume and prothrombotic state play a predisposing role. In addition, estrogen is related to an increase in matrix mucopolysaccharides, smooth muscle hypertrophy, and decreased collagen production. In connective tissue diseases, the coronary arterial wall is weakened by medial degeneration [15].

Based on the concept of vulnerability of coronary vessel walls, we propose that the presence of defective type IV collagen represents a predisposing factor which when combined with localized shearing forces to the coronary wall produced by fluctuating and uncontrolled hypertension may lead to intimal tearing of the coronary arteries and the pathogenesis of SCAD in AS. However, this remains speculative as it is unsafe to obtain coronary artery biopsies in human subjects. Future studies with coronary artery biopsy in mice model with* COL4A5* gene mutation would be helpful to further investigate this proposed mechanism.

At this time there is no consensus on treatment guidelines for SCAD. The treatment options include a conservative approach versus revascularization with percutaneous coronary intervention (PCI) or coronary artery bypass grafting [13, 14]. A decision regarding the management should be based on the severity of the presentation, evidence of ongoing ischemia or infarction, and the risk of possible PCI-related complications [16]. The patient we present was treated with a drug eluting stent and has been without complications for three years.
